# eLearning among Canadian anesthesia residents: a survey of podcast use and content needs

**DOI:** 10.1186/1472-6920-13-59

**Published:** 2013-04-23

**Authors:** Clyde T Matava, Derek Rosen, Eric Siu, Dylan M Bould

**Affiliations:** 1 Department of Anesthesia and Pain Medicine, Hospital for Sick Children, Toronto, ON, Canada; 2 Department of Anesthesia, Faculty of Medicine, University of Toronito, Toronto, Ontario, Canada; 3 Department of Anesthesia, Toronto General Hospital, University Hospital Network, Toronto, Ontario, Canada; 4 Faculty of Medicine, University of Toronto, Toronto, Ontario, Canada; 5 Department of Anesthesiology, Children’s Hospital of Eastern Ontario, University of Ottawa, Ottawa, ON, Canada

**Keywords:** Podcasts, Residents, e-learning, Survey, Needs, Content, Anesthesia

## Abstract

**Background:**

Podcasts are increasingly being used in medical education. In this study, we conducted a survey of Canadian anesthesia residents to better delineate the content needs, format preferences, and usage patterns among anesthesia residents.

**Methods:**

10/16 Canadian anesthesia program directors, representing 443/659 Canadian anesthesia residents, allowed their residents to be included in the study. 169/659 (24%) residents responded to our survey. A 17-item survey tool developed by the investigators was distributed by email eliciting information on patterns of podcast use, preferred content, preferred format, and podcast adjuncts perceived to increase knowledge retention.

**Results:**

60% (91/151) had used medical podcasts with 67% of these users spending up to 1 hour per week on podcasts. 72.3% of respondents selected ‘ability to review materials whenever I want’ was selected by the majority of respondents (72%) as the reason they found podcasts to be valuable. No clear preference was shown for audio, video, or slidecast podcasts. Physiology (88%) and pharmacology (87%) were the most requested basic science topics while regional anesthesia (84%), intensive care (79%) and crisis resource management (86%) were the most requested for procedural, clinical and professional topics respectively. Respondents stated they would most likely view podcasts that contained procedural skills, journal article summaries and case presentations and that were between 5-15 minutes in duration A significantly greater proportion of senior residents (81%) requested podcasts on ‘pediatric anesthesia’ compared to junior residents 57% (*P* = 0.007).

**Conclusions:**

The majority of respondents are using podcasts. Anesthesia residents have preferred podcast content, types, length and format that educators should be cognizant of when developing and providing podcasts.

## Background

Podcasting is a technology that allows for the distribution of video and audio recordings (podcasts) over the internet as digital media files. These podcasts can be viewed online or downloaded to a user’s computer or handheld multimedia device (i.e. portable mp3 player, smart phone, tablet device) and carried with them [1]. Podcasts are increasingly being used in medical education [2-5]. They have the key advantage that the presentation of information does not have to be linked with any particular time or location [6,7]. They can be a significant learning aid to auditory learners (audio podcasts) and visual learners (video podcasts) for distance education [7-9]. In addition to inexpensive production costs, podcasts also confer high ease of use, rapidity of deployment, and offer the opportunities to share information around the world [8,10-13]. Finally, podcasts may be better suited than lectures to the learning styles of “Generation Y” medical students and residents [1,14-18].

A major limitation of achieving and maintaining high user rates in podcasting is the development of high quality content that matches the needs of the targeted user-group [4,13]. To better delineate these needs and usage patterns, we conducted a survey of Canadian anesthesia residents. We hypothesized that anesthesia residents have clear podcast content needs and usage patterns which may depend on the year of training.

## Methods

Following approval by the Research Ethics Board at Children’s Hospital of Eastern Ontario, we contacted 16 anesthesia residency program directors for permission to recruit their residents into the study. We defined junior residents as post-graduate year 1-3 (PGY 1-3) and senior residents as PGY 4-5.

### Survey design

A review of the literature and commonly used textbooks was conducted in order to identify anesthesia topics for inclusion in a 17-item survey tool. The survey was constructed using the online tool, Qualtrics, at http://www.qualtrics.com (Provo, UT, USA) using published guidelines for the creation of online surveys [19-21]. Following pretesting by the authors and pilot testing on anesthesia fellows, the final survey tool was translated into French, followed by further pilot testing of the French version. Options for both English and French translations were provided to all respondents. The final survey tool (Additional file 1) elicited information on residents’:

1. Current patterns of podcast use;

2. Preferred content (divided into 4 categories: basic sciences, procedural, clinical and professional topics);

3. Preferred podcast formats (duration and content type);

4. Podcast adjuncts perceived to increase knowledge retention.

### Survey distribution and collection

Our sampling frame consisted of all residents enrolled in anesthesia residency programs across Canada for the 2010-2011 academic year. E-mail invitations were sent to anesthesia program directors’ offices asking them to forward these emails to their residents. Three reminder emails were sent to program directors, each a month apart. As an incentive, residents were invited to enter into a prize draw for an iPad™(Apple, Cupertino, CA) on completion of the survey. All responses were kept anonymous and delinked from identifying information. All responses were stored on http://www.qualtrics.com and exported to an Excel (Microsoft, Redmond, WA) spreadsheet for analysis. Consent for participation in the study was implied by completion of the survey.

### Data analysis

We analysed the perceived needs in podcast content of all respondents and compared the data of junior residents to senior residents. Data analysis was performed using Prism version 5.0b for Mac OS X (GraphPad Software, San Diego, CA). Descriptive statistics were used to summarize the data. and two-tailed Fisher’s exact test was used to analyze nominal data. A value of P < 0.05 was considered to indicate statistically significant differences.

## Results

### Demographic and current patterns of use

10/16 anesthesia program directors, representing 443 residents, gave permission for their residents to be included in the study. 216 residents in the remaining 6 programs did not participate in the study. 169 residents responded (38% response rate).

**Figure 1:**
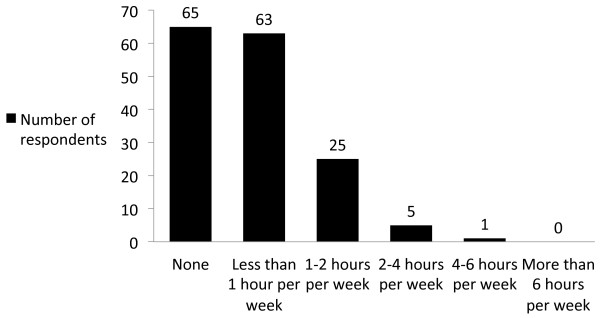
Hours spent on podcasts per week by respondents.

Sixty percent (91/151) reported using medical podcasts (Table 1), with 67% of users spending up to 1 hour per week on podcasts (Figure 1). Accessing (streaming) podcasts online via a computer 45% (73/159) and downloading to a portable media device (mp3 player, smartphone, tablet) 38% (61/159) were both popular methods for reviewing podcasts (Table 2). Almost half of respondents would use podcasts as part of routine study while less than a third would use them prior to a case in the Operating room (OR)/clinic/Intensive Care Unit (ICU) (Table 2). The majority of respondents selected ‘ability to review materials whenever I want’ (72%) and ‘…wherever I want’ (66%) as reasons why they found podcasts to be valuable. Respondents showed no clear preference when asked which of audio, video, or slidecasts (podcasts with audio and still images or Microsoft Powerpoint slides) best suited their learning styles (Table 2).

**Table 1:** Proportions of podcast users and non-users

		**Non-users of podcasts**	**Podcast users**
Gender (M/F)*		31/29 (52/48)	54/37 (59/41)
Total		60 (40)	91 (60)
Year of Training †			
	PGY 1	17 (29)	26 (31)
	PGY 2	10 (18)	15 (18)
	PGY 3	9 (14)	21 (25)
	PGY 4	8 (14)	13 (16)
	PGY 5	13 (23)	9 (11)
	Total	57 (100)	84 (100)

Data are *n* (percentage).

N = 159.

* 151 responses, 8 respondents skipped question.

† 141 responses, 18 respondents skipped question.

**Table 2:** Podcast use characteristics of all respondents

		**Respondents N = 159 (%)**
*Methods used to view podcasts*		
	View (stream) online via computer	71 (45)
	Download material to a handheld device (iPod/other MP3 player/smartphone/tablet device) to watch later	61 (38)
	Download material onto computer	53 (33)
	View (stream) online via handheld device (iPod/mp3 Smartphone/tablet device)	34 (21)
*Manner in which podcasts are currently used*		
	Part of routine study	76 (48)
	To introduce a new topic	60 (38)
	Revision for exam	33 (21)
	Preview prior to case in the OR/Clinic/ICU	29 (18)
*Features of podcasts found to be valuable by respondents*		
	Ability to review materials whenever I want	115 (72)
	Ability to review materials wherever I want	113 (71)
	Ability to review materials at my own pace	105 (66)
	Ability to review materials repeatedly	85 (51)
*Podcasts best suited to respondents’ personal learning style*		
	Podcasts with audio and still images or powerpoint slides	59 (37)
	Audio podcasts	54 (34)
	Video podcasts	45 (28)

Data are *n* (percentage).

The majority of respondents, 98% (58/60), who do not use podcasts selected ‘I did not know they were available’ as the reason for non-use. Only 11.7% (7/60) of non-users did not use podcasts because of ‘no access’ to an iPod / MP3 player/ smartphone (Table 3).

**Table 3:** Reasons for non-use of podcasts

**Reason selected**	**Responses**
I did not know they were available	58 (97)
I am not used to accessing course materials via podcast.	28 (47)
I don’t have enough time to watch/listen to a podcast	12 (20)
The quality of the information in the podcasts is poor	9 (15)
I do not have an access to an iPod / a MP3 player/ smartphone.	7 (12)
I experienced technical problems	7 (12)
I do not like accessing course materials via podcast.	4 (7)

Data are *n* (percentage).

N = 60.

### Preferred podcast content

**Figure 2:**
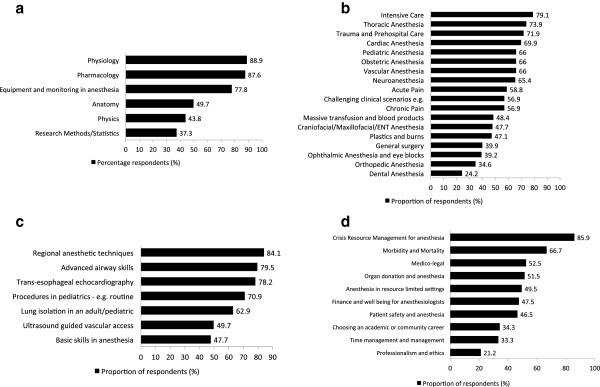
**Rankings of content topics by respondents. a**. Ranking of basic science topics by respondents (%). **b**. Ranking of clinical topics by respondents (%). **c**. Ranking of procedural topics by respondents (%). **d**. Ranking of professional topics by respondents (%).

Physiology (89%) and pharmacology (88%) were the most requested basic science topics by all respondents (Figure 2A). The most requested clinical topics included intensive care (80%) and thoracic anesthesia (74%) (Figure 2B). Under the procedural topic category, respondents requested podcasts on regional anesthesia (84%) and advanced airway skills (80%) (Figure 2C), while crisis resource management (86%), and morbidity/mortality (67%) were most requested for the professional category (Figure 2D). Research methods/statistics (37%) and dental anesthesia (24%) were the least popular topics for podcasts.

There were noticeable differences in perceived podcast topic need by level of training. A significantly greater proportion of senior residents (81%) requested podcasts on ‘pediatric anesthesia’ compared to junior residents 57% (*P* = 0.007) (Table 4). Similarly, greater proportions of senior residents compared to junior residents requested podcasts on ‘thoracic anesthesia‘ and ‘mortality and morbidity in anesthesia’, however, these were not statistically significant (Table 4).

**Table 4:** Preferred podcast topics* by training level

	**Junior residents†**	**Senior residents‡**
*Basic science topics*		
Physics	49 (50)	17 (40)
Physiology	86 (88)	38 (88)
Anatomy	47 (48)	23 (56)
Pharmacology	87 (89)	36 (84)
Research Methods/Statistics	37 (39)	15 (35)
Equipment and monitoring in anesthesia	77 (79)	33 (77)
Basic skills in anesthesia, e.g. rapid sequence induction, basic airway skills, prone positioning ,TIVA	45 (47)	17 (40)
*Procedural topics*		
Regional anesthetic techniques	78 (81)	36 (84)
Ultrasound guided vascular access	52 (54)	16 (38)
Advanced airway skills, e.g. jet ventilation, intubating laryngeal mask, fiber-optic techniques, cricothyroidotomy, surgical airway.	74 (77)	33 (78)
Procedures in pediatrics - e.g. routine and difficult pediatric airway, regional techniques, difficult vascular access	69 (72)	28 (65)
Lung isolation in an adult/pediatric patient	59 (62)	26 (61)
Trans-esophageal echocardiography	77 (81)	32 (74)
Acute Pain	58 (59)	24 (56)
*Clinical topics*		
Chronic Pain	55 (56)	28 (65)
Obstetric Anesthesia	63 (64)	29 (68)
Vascular Anesthesia	62 (63)	29 (67)
Ophthalmic Anesthesia and eye blocks	39 (40)	16 (37)
Trauma and Prehospital Care	69 (70)	30 (70)
Cardiac Anesthesia	68 (69)	28 (65)
Thoracic Anesthesia	67 (68)	36 (84)
Neuroanesthesia	59 (60)	32 (74)
Plastics and burns	46 (47)	20 (47)
Orthopedic Anesthesia	32 (33)	16 (38)
Pediatric Anesthesia	56 (57)	35 (81)¶
General surgery, gynaecology and urology (including laparoscopy and anesthesia)	40 (49)	15 (35)
Intensive Care	70 (71)	38 (88)
Craniofacial/Maxillofacial/ENT Anesthesia	46 (47)	17 (40)
Dental Anesthesia	24 (24)	11 (27)
Challenging clinical scenarios e.g. malignant hyperthermia, morbid obesity, childhood syndromes	88 (90)	37 (86)
Massive transfusion and blood products	73 (75)	34 (79)
*Professional topics*		
Professionalism and ethics	21 (21)	9 (21)
Patient safety and anesthesia	45 (46)	18 (42)
Medico-legal issues and anesthesia	49 (50)	18 (42)
Mortality and morbidity in anesthesia	58 (59)	33 (77)
Time management and anesthesia	35 (36)	14 (33)
Crisis management and anesthesia	82 (84)	35 (82)
Anesthesia in the resource limited setting	50 (51)	21 (49)
Choosing between academic and non academic careers	45 (46)	12 (28)
Organ donation and anesthesia	47 (48)	20 (47)
Finance and well being for anesthesiologists	54 (55)	19 (44)
Total	98 (100)	43 (100)

Data are *n* (percentage).

*18 respondents skipped this question.

†PGY 1 - 3.

‡PGY 4-5.

¶ P = 0.007.

A significantly greater proportion of male respondents (63%) preferred podcasts on ‘ultrasound guided vascular access’ compared to female respondents (34%) (*P* = 0.0005). Similarly significantly more male respondents (55%) versus female respondents (35%) preferred podcasts content on ‘Basic skills in anesthesia, e.g. rapid sequence induction, basic airway skills, prone positioning and TIVA’ (*P* = 0.01).

### Preferred podcast formats

**Figure 3:**
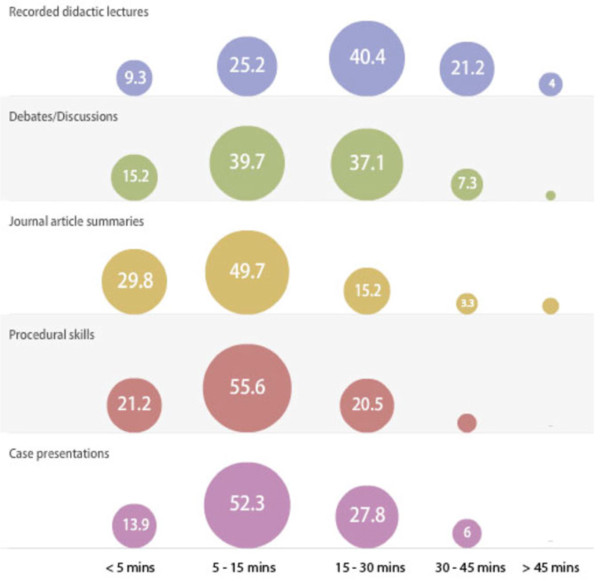
**Preferred podcast duration by content type.** (Data are in percentage of respondents).

Respondents demonstrated a preference for podcasts containing procedural skills, journal article summaries and case presentations, that were of 5-15 minutes (min) duration (Figure 3). Didactic lecture podcasts of 15-30 min duration were more likely to be viewed compared to either shorter podcasts or those longer than > 45 min (Figure 3). For all other categories, respondents preferred shorter podcasts of 5-15 minutes and were particularly less likely to view podcasts of > 45 min (Figure 3). Practice oral exam questions were very popular among residents, with 25% responding that they would be ‘likely’ and 67% ‘very likely’ to watch these podcasts (Table 5). Residents also perceived the inclusion of pre-, and post-test multiple-choice questions (MCQs) in podcasts to be effective for knowledge retention (Table 6).

**Table 5:** Percentage of respondent’s likelihood of watching a podcast type (N = 151)*

	**Podcast type**	**Don’t know**	**Very unlikely**	**Unlikely**	**Likely**	**Very likely**
1	Recorded didactic lectures	5	5	11	48	30
2	Debates/discussions	10	13	21	46	11
3	Journal summaries	5	4	17	52	21
4	Procedural skills	4	3	11	45	37
5	Case presentations	3	2	11	61	23
6	Practice oral exams	3	1	5	25	67

Data are shown as percentage only for clarity.

*8 respondents skipped question.

**Table 6:** **Methods perceived by residents to be effective for facilitating knowledge retention during podcast us**e

**Method(s) for facilitating knowledge retention during podcast use**	**Respondents N = 159 (%)**
Scheduled online chat	4 (3)
Discussion board posts	11 (7)
Links to articles	37 (23)
Pre-post test MCQ	108 (68)

Data are *n* (percentage).

## Discussion

We have demonstrated that the majority of respondents (60%) use podcasts. Anesthesia residents appear to have well defined podcast content preferences. Furthermore, anesthesia residents have preferred podcast types, length and format, along with a preference for podcasts to incorporate multiple-choice questions.

In the absence of prior work on podcast use among medical residency trainees, our study provides initial data on podcast use and preferred content among anesthesia residents. Residents’ preferences on content, duration and format should be considered by podcast creators and program directors for developing new material that better suits the needs of trainees.

Whilst 60% of anesthesia residency respondents using medical podcasts does suggest a high uptake of this relatively new technology, it is slightly lower than the 71.2% usage reported by Fietze et al in a survey of Faculty of Economics and Social Studies students at the University of Flensburg, Germany [22]. This may reflect a difference in the update and use of technology between countries or disciplines. There are no similar published studies of podcast use among medical trainees. The relatively high use of podcasts in our study may be explained in part by the proliferation of mobile devices, such as internet-ready smartphones, that support both audio and video files. Thirty-eight percent of the residents accessed podcasts on a mobile device (smartphone, iPod, mp3 players, etc.). Other advances in technology which may have contributed to higher rates of podcasts use include the wider availability of high-speed internet, wireless network-capable tablet devices and greater computer literacy among medical personnel.

Half of the anesthesia residents in this current study reported using podcasts as part of routine studying, while under a fifth used them for learning prior to a case in the OR/clinic/ICU. The level of podcasts use for just-in-time learning in our study appears to be low when compared to levels of use reported among health care students [3,6,23]. The existence of relevant podcast topics may result in an increase of podcasts as just-in-time learning.

There was a surprising finding of gender based differences in podcast topics focused on technical skills. Male respondents preferred podcast content on the use of ultrasound guided vascular access. This finding is not easily explained by our study and warrants further studies investigating gender based preferences on techincal procedures among anesthesia trainees and consultant level anesthesiologists.

Our study also demonstrated that there was no clear preference in podcast type. Podcasts with video, slideshows, or just audio podcasts were equally popular. While investigating specific respondents’ specific learning styles was out of the scope of our study, previous work looking at learning styles among anesthesia residents suggested that anesthesia trainees tend to have an aptitude for auditory and visual learning, which may be well complemented with audio-visual podcasts [14-17]. Furthermore, anesthesia video podcasts may also provide training in procedural skills, which are a key component in anesthetic practice. Respondents in our study also selected commonly cited advantages for podcast use, such as portability and ease of accessibility, as reasons they find them valuable [6,7,24-26].

An important finding in our study is that residents have preferred podcast content topics (Table 2). These preferences exist across the spectrum of categories from basic sciences, procedural and clinical, to professional and ethical topics. Topics received similar ratings by both junior and senior residents. A surprising finding was the popularity of basic science topics such as physiology and pharmacology. Physiology and Pharmacology are traditionally taught via didactic sessions. Residents may prefer these topics to be offered via podcasts as they can be readily available for revision. Our study did not explore whether residents would prefer to completely abandon traditional didactic lectures in preference of podcasts.

Residents in this study selected highly complex procedural skill topics such as regional anesthesia and difficult airway as preferred content for podcasts. This is not surprising as procedural skills are core competencies required of anesthesiologists and anesthesia trainees [27]. Recent studies have highlighted the superior nature of video in the teaching and learning of procedural skills over traditional paper based methods for surgical residents and undergraduate students [1,25,28]. In essence, it is easier to learn a new procedure or review one by watching a video demonstration rather than by reading about it on paper [29].

The training level of respondents seemed to dictate relatively few differences in topic preferences. Senior residents chose pediatric anesthesia content more frequently than junior residents. This may be because junior residents have not yet been exposed to pediatric patients and are less familiar with this course material, whilst senior residents may feel the need to have more information on this topic to supplement their regular lectures. Similarly, senior residents preferred crisis management as well as morbidity and mortality in anesthesia as professional topics for podcasts. This may reflect their greater exposure to these issues in the operating room and their awareness of the importance of a reflective practice as they near the completion of their training.

Our study demonstrated that residents are more likely to view podcasts of procedural skills, journal article summaries of 5-15 min duration and recorded didactic lecture podcasts of 15-30 min. This may be explained by the fact that respondents perceive that a certain amount of time is necessary to convey key learning objectives. This data will guide podcasts creators to meet the needs of residents in podcasting. Interestingly, 92% percent of respondents were ‘likely‘ or ‘very likely‘ to watch practice oral exam podcasts. This is an obvious area for development and it is likely these podcasts would prove popular as they allow for exam-focused studying amongst medical trainees [30]. Residents also perceived the use of multiple-choice questions to be effective for knowledge retention. This is supported by recent studies that demonstrate that teaching with the ‘testing effect’ enhances knowledge retention and the finding that residents would prefer this feature is encouraging for the practical implementation of this evidence-based medical education [31]. Podcast producers may incorporate this by having audio or video pre- and post-exposure MCQs spliced into podcasts.

Some methodological issues limited our study. Firstly, non-response bias is common and unavoidable with anonymized electronic surveys [32]. We acknowledge that by completing an online survey on medical podcasts, our respondents have demonstrated a certain level of computer literacy and podcast awareness that may have made them more likely to respond to the survey. Furthermore, non-response bias may have been exaggerated by the incentive of an iPad™ as this would appeal to those who enjoy use of such technology. It is also possible that those residents who did not use podcasts were therefore less familiar with this topic and so did not respond to the survey. Secondly, the response rate of 38% was relatively low. However, this response rate is comparable to the response rates of previously published surveys among health professionals, whose response rates have ranged from 26%-46% [32,33]. Our response rate may be explained in part by survey fatigue as medical trainees are increasingly inundated with multiple surveys resulting in lower response rates [34].

## Conclusions

Our study is the first to report podcast content needs and preferred formats among specialty trainees. Anesthesia residents appear to have well defined podcast content preferences. In addition, senior and junior anesthesia residents generally have similar podcast content needs with differences in a few categories. Anesthesia residents also report preferred podcast type, length and format for specific content. This allows educators to produce training level specific podcast material. This podcast needs assessment may help inform podcast creators and program directors on content and formats for podcast development.

## Competing interests

The authors declare that they have no competing interests.

## Authors’ contributions

CTM designed the study. CTM and DR conducted the study, collected and analyzed the data. CM wrote the manuscript. DR, ES, DMB reviewed the manuscript. All authors read and approved the final manuscript.

## Pre-publication history

The pre-publication history for this paper can be accessed here:

http://www.biomedcentral.com/1472-6920/13/59/prepub

## Supplementary Material

Additional file 1 **Survey tool.** The survey used for this study is attacehd as a separate file. Click here for file
