# Supplementary material for: eLearning among Canadian anesthesia residents: a survey of podcast use and content needs
Source: BMC Med Educ. 2013 Apr 23;13:59. doi: 10.1186/1472-6920-13-59 (PMC3648490; doi:10.1186/1472-6920-13-59)
Supplement: Additional file 1 — Survey tool. The survey used for this study is attacehd as a separate file. [file 1472-6920-13-59-S1.pdf]

**Appendix 1. Survey Tool**

A Nationwide Survey: Canadian Anesthesia Residents' Podcasts Needs

Intro A Nationwide Survey: Canadian Anesthesia Residents' Podcasts Needs
Thank you for agreeing to complete this important survey on the podcast content needs of residents training in Canada. All responses are non-identifiable. By completing this survey you are agreeing to the use of the results in future publications. On completion of the survey you are eligible to enter a draw to win an iPad by entering your email address at the end of the survey. You are free to leave any question blank that you are uncomfortable with.This study has been approved by the CHEO Research Ethics Board #11/57X.Thank you for your time.

Q1 How many hours per week do you spend viewing or listening to medical podcasts?

- None
- Less than 1 hour per week
- 1-2 hours per week
- 2-4 hours per week
- 4-6 hours per week
- More than 6 hours per week

Q2 Indicate the method(s) you use to review the podcast materials.

- View (stream) material on a computer online
- Download material onto a computer to watch later
- View (stream) material on a handheld device (iPod/other MP3 player/smartphone/tablet device)
- Download material to a handheld device (iPod/other MP3 player/smartphone/tablet device) to watch later

Q3 Which of the following describe how you use podcasts? (Select all that apply)

- Preview prior to a case in the OR/ICU/Clinic
- Revision for exam
- Part of your routine study
- To introduce a new topic
- Other ____________________

Q4

Which of the following podcast formats best suits your personal learning style?

- Audio podcasts
- Podcasts with audio and still images or powerpoint slides
- Video podcasts

Q5 Which of the following features of podcasts do you find most valuable? (select all that apply)

- Ability to review materials wherever I want.
- Ability to review materials whenever I want.
- Ability to review materials at my own pace.
- Ability to review materials repeatedly.
- Other ____________________

Q6 Which of the following basic science/theoretical background topics would you like to see available in podcasts? (Select all that apply)

- Physics
- Physiology
- Anatomy
- Pharmacology
- Research Methods/Statistics
- Equipment and monitoring in anesthesia

Q7 Which of the following procedural topics would you like to see available in podcasts? (Select all that apply)

- Basic skills in anesthesia, e.g. rapid sequence induction, basic airway skills, prone positioning ,TIVA
- Regional anesthetic techniques
- Ultrasound guided vascular access
- Advanced airway skills, e.g. jet ventilation, intubating laryngeal mask, fiber-optic techniques, cricothyroidotomy, surgical airway.
- Procedures in pediatrics - e.g. routine and difficult pediatric airway, regional techniques, difficult vascular access
- Lung isolation in an adult/pediatric patient
- Trans-esophageal echocardiography

Q8 Which of the following clinical topics would you like to see available in podcasts? (Select all that apply)

- Acute Pain
- Chronic Pain
- Obstetric Anesthesia
- Vascular Anesthesia
- Ophthalmic Anesthesia and eye blocks
- Trauma and Prehospital Care
- Cardiac Anesthesia
- Thoracic Anesthesia
- Neuroanesthesia
- Plastics and burns
- Orthopedic Anesthesia
- Pediatric Anesthesia

- General surgery, gynaecology and urology (including laparoscopy and anesthesia)
- Intensive Care
- Craniofacial/Maxillofacial/ENT Anesthesia
- Dental Anesthesia
- Challenging clinical scenarios e.g. malignant hyperthermia, morbid obesity, childhood syndromes
- Massive transfusion and blood products

Q9 Which of the following professional topics would you like to see available in podcasts? (Select all that apply)

- Finance and well being for anesthesiologists
- Professionalism and ethics
- Patient safety and anesthesia
- Medico-legal issues and anesthesia
- Mortality and morbidity in anesthesia
- Time management and anesthesia
- Crisis management and anesthesia
- Anesthesia in the resource limited setting
- Choosing between academic and non academic careers
- Organ donation and anesthesia

Q10 What is your preferred length for each of the following types of podcast?

| | < 5 mins | 5-15 mins | 15-30 mins | 30-45 mins | > 45 mins |
|---|---|---|---|---|---|
| Recorded didactic lectures | ❍ | ❍ | ❍ | ❍ | ❍ |
| Debates/Discussions | ❍ | ❍ | ❍ | ❍ | ❍ |
| Journal article summaries | ❍ | ❍ | ❍ | ❍ | ❍ |
| Procedural skills | ❍ | ❍ | ❍ | ❍ | ❍ |
| Case presentations | ❍ | ❍ | ❍ | ❍ | ❍ |

Q11 To what extent are you likely to download or view online the following podcast types of acceptable time length?

| | Don't Know | Very Unlikely | Unlikely | Likely | Very Likely |
|---|---|---|---|---|---|
| Recorded didactic lectures | ❍ | ❍ | ❍ | ❍ | ❍ |
| Debates/Discussions | ❍ | ❍ | ❍ | ❍ | ❍ |
| Journal summaries | ❍ | ❍ | ❍ | ❍ | ❍ |

| | | | | | |
|---|---|---|---|---|---|
| Procedural skills | ❍ | ❍ | ❍ | ❍ | ❍ |
| Case presentations | ❍ | ❍ | ❍ | ❍ | ❍ |
| Practice Oral Exams | ❍ | ❍ | ❍ | ❍ | ❍ |

Q12 Which of the following do you think would be the most useful method to enable retention of knowledge gained from podcasts?

- Scheduled live online chat
- Discussion board posted alongside the podcast
- Links to articles which cover the topic in more depth
- Pre- and Post- MCQs
- Other ____________________

Q13 If you do not use podcasts, which of the following reasons best explain(s) why not? (Select all that apply)

- I do not have an access to an iPod / a MP3 player/ smartphone.
- I did not know they were available
- I am not used to accessing course materials via podcast.
- I do not like accessing course materials via podcast.
- I experienced technical problems
- I don't have enough time to watch/listen to a podcast
- The quality of the information in the podcasts is poor
- Other ____________________

Q14 What is your year of training?

- PGY 1
- PGY 2
- PGY 3
- PGY 4
- PGY 5
- Family Practice (GP) Anesthesia
- Dental Anesthesia
- Other ____________________

Q15 What is your gender?

- Male
- Female

Q16 Select your university

- University of British Columbia

- University of Calgary
- University of Alberta
- University of Saskatchewan
- University of Manitoba
- University of Western Ontario
- McMaster University
- University of Toronto
- Queen's University
- University of Ottawa
- McGill University
- Université de Montréal
- Université de Sherbrooke
- Université Laval
- Dalhousie University
- Memorial University of Newfoundland
- Northern Ontario School of Medicine

Q17 Please enter any additional comments you would like to make

Q18 To be entered into the draw to win an iPad, please enter your email address below and then hit the "Submit" button.

Enter your email address in the box
